# Supplementary material for: Deletion of Crtc1 leads to hippocampal neuroenergetic impairments associated with depressive-like behavior
Source: Mol Psychiatry. 2022 Oct 12;27(11):4485–501. doi: 10.1038/s41380-022-01791-5 (PMC9734042; doi:10.1038/s41380-022-01791-5)
Supplement: Supplementary file 1 — Supplemental material [file 41380_2022_1791_MOESM1_ESM.docx]

## **Supplement**

## Supplemental Materials and Methods

## Western blot analysis

Hippocampal proteins were extracted with AllPrep DNA/RNA/Protein Mini kit (Qiagen, Venolo, Netherland). Protein quantification was done with the Bradford Assay (BioRad). Ten μg of tissue homogenates were diluted 1:1 with sample buffer [50 mM Tris HCl (pH 6.8), 100 mM DTT, 2% SDS, 9% Glycerol, 1% Bromophenol Blue], separated on a 10% SDS-polyacrylamide gel, and proteins were transferred to polyvinylidene difluoride (PVDF) membranes with the Trans-Blot Turbo Transfer system (BioRad, Hercules, CA, USA). Total proteins were stained with the Pierce Reversible Protein Stain Kit for PVDF membranes (Thermo Scientific) and imaged by the Fusion Solo S documentation system (Vilber). After destaining, blots were blocked for 2 hours at room temperature (RT) in TBST [10 mM Tris-HCl (pH 7.4), 150 mM NaCl, 0.1% Tween-20], supplemented with 5% skim milk powder, and then incubated with a primary antibody in TBST plus 5% skim milk powder overnight at 4°C. After three 10-min washes in TBST, PVDF membranes were incubated for 1 hour at RT with horseradish peroxidase (HRP)-conjugated secondary antibodies in TBST plus 5% skim milk powder, washed 3 times in TBST, and developed using a SuperSignal West Pico Plus Chemiluminescence detection kit (Thermo Scientific). The following antibodies and dilutions were used: rabbit α-Creatine kinase B type (CKB) 1:10’000 (ab92452, abcam, Cambridge, UK), rabbit α-Creatine kinase MT (CKMT) 1:1000 (ab198257, abcam, Cambridge, UK), Goat α-rabbit-HRP 1:10’000 (EMD Millipore, USA). Chemiluminescence was detected and imaged with the Fusion Solo S documentation system (Vilber). Quantification of band intensity was performed with Image J software (National Institute of Health, Bethesda, MD, USA). CKB and CKMT band intensities were normalized with total protein signals.

## Chromatin Immunoprecipitation with sequencing (ChIP-seq)

Hippocampi from twelve 8-week-old C57BL/6N male mice were quickly collected and flash-frozen in a single tube that was sent to Actif Motive Inc. (Carlsbad, CA, USA) for analysis. Fixation, chromatin extraction and immunoprecipitation was performed according to the company’s procedures. Two ChIP reactions were carried out using 30 μg of chromatin and anti-CRTC1 antibodies from Cell Signaling Technology (CST, cat# 2587) and Bethyl Labs (cat# A300-769A). The ChIP DNAs were processed into standard Illumina ChIP-Seq libraries and sequenced to generate >5 million reads. Reads were aligned to the mouse genome (mm10), and after removal of duplicate and non-uniquely mapped reads, ~5.5 and ~6.1 million alignments were obtained for the CST and Bethyl sample, respectively. Signal maps capturing fragment densities along the genome were generated and visualized in the Integrated Genome Browser (IGB). In addition, MACS peak finding was performed to identify the most significant peaks. Using a default cutoff of p-value 1e-7 (without control file), 10,159 and 8,449 peaks were identified for the CST and Bethyl assays, respectively (after ENCODE blacklist filtering).

## Supplemental Figures

## Supplemental Figure 1

**Figure S1: Deletion of *Crtc1* in mice is associated with increased inflammatory markers in PFC as revealed by MRS**

**(A)** T_2_-weighted image acquired for localized MRS (VOI including cingulate PFC: yellow rectangle) with a scale bar of 2mm (left) and typical ^1^H-MRS spectrum acquired in the VOI (right). **(B)** Quantification of PFC neurochemical profile in 6 weeks old wild-type (WT; n=10) and *Crtc1^-/-^* (n=6) mice, *** *P*<0.005, **P*<0.05, unpaired Student’s t-test. **(C)** Volumetric analysis of MRI images reveals a higher prefrontal volume in *Crtc1^-/-^* as compared to wild-type mice (****P*<0.005, ** *P*<0.01, * *P*<0.05, unpaired Student’s t-test). **(D)** Relative *Crtc1* expression is not significantly different between PFC and dorsal hippocampus (DH) in wild-type mice (unpaired Student’s t-test, n.s., not significant). Data are shown as mean±s.e.m.

## Supplemental Figure 2

**Figure S2: Supplementary data of the longitudinal experiment (6 to 24 weeks of age)**

**(A)** Relative gene expression in hippocampus of wild-type (WT; n=10) and *Crtc1^-/-^* (n=6) mice after the 18 weeks of social isolation. *Glut1-4*, Glucose transporter 1-4*; Mct1-4*, monocarboxylate transporter 1-4; *Pfk1*, phosphofructokinase 1; *Pfkfb2*, 6-phosphofructo-2-kinase/fructose-2,6-biphosphatase 2. **(B)** Locomotor activity of wild-type (n=10) and *Crtc1^-/-^* (n=6) mice during the longitudinal isolation experiment. **(C)** Total choline (tCho; GPC+PCho) profile in PFC of wild-type (n=10) and *Crtc1^-/-^* (n=6) mice during the isolation protocol. tCho remained increased in *Crtc1^-/-^* independently of animal’s age (left panel; Genotype effect: F_1,14_=12.89, *P*<0.01; Time effect: F_2,28_=29.31, *P*<0.0001, two-way ANOVA, followed by Fisher LSD posthoc test; ****P*<0.005, ***P*<0.01, **P*<0.05; # refers to wild-type only).

## Supplemental Figure 3

**Figure S3: Volumetric analyses of prefrontal volume during the longitudinal experiment (6 to 24 weeks of age)**

**(A)** Typical T_2_-weighted images analyzed with location of the ROI drawn on PFC (red kite); Yellow scale bar=2mm; white label: bregma coordinates (mm). **(B-D)** Prefrontal (left panels) and ventricular (right panels) volumes measured from MRI images at the age of 6 weeks (b), 12 weeks (c) and 24 weeks (d). Unpaired Student’s t-test, ****P*<0.005, ***P*<0.01, **P*<0.05, n.s., not significant. **(E)** Whole brain volume measured from MRI images at the age of 6 weeks. **(F)** Correlation between PFC total choline (tCho: GPC+PCho) and PFC volume (R=0.31, **P*=0.03).

## Supplemental Figure 4

**Figure S4: Supplementary hippocampal gene expression analyses and prefrontal volumetric analyses after ebselen treatment and OSFST.**

**(A)** Hippocampal gene expression analysis after 21 days of ebselen treatment. (*Pfkfb2*: Interaction: F_1,28_=4.87, ^§^*P*=0.037, two-way ANOVA). *Pfk1*, phosphofructokinase 1; *Pfkfb2*, 6-phosphofructo-2-kinase/fructose-2,6-biphosphatase 2.; *Glut1-3*, Glucose transporter 1-3; *Mct1-4*, monocarboxylate transporter 1-4. **(B)** Western blot analysis of creatine kinases after 21 days of ebselen treatment. Mitochondrial type was increased in *Crtc1^-/-^* mice (U-MtCK: Genotype effect: F_1,23_=25.06, *P*<0.0001; two-way ANOVA, followed by Bonferroni’s test, **P*=0.027, ****P*=0.0005), while cytoplasmic type was only slightly affected by treatment (B-CK: Treatment effect: F_1,23_=5.043, *P*=0.035; two-way ANOVA, followed by Bonferroni’s test, **P*=0.022). **(C)** Prefrontal volumetric analysis from neuroanatomical MRI images at baseline day -10; Scan 1), before the start of the treatment (day -1; Scan 2) and at the end of the protocol (day 21) between the treated (EBS) and untreated (VEH) groups. Unpaired Student’s t-test, ****P*<0.005, ***P*<0.01, **P*<0.05, n.s., not significant. **(D)** Prefrontal total choline concentration (tCho; GPC+PCho; left panel) at the end of the treatment (Genotype effect: F_1,28_=26.16, *****P*<0.0001, two-way ANOVA). Prefrontal volume (right panel) at the end of the treatment (Genotype effect: F_1,32_=4.21, **P*<0.05, two-way ANOVA). **(E)** Correlation between PFC total choline (tCho: GPC+PCho) and PFC volume (R=0.35, **P*=0.0005). **(F)** Receiver operating characteristic (ROC) curves for prefrontal cortex (PFC) total choline (tCho) concentration (green), volume (blue) and the average of the z-scores of both measurements (tCho+Volume; black). Using a combination of both cholinergic and volumetric markers (averaged z-scores) provided an area under the curve (AUC) of 0.820 (95% CI 0.754-0.886), thus providing a good means of distinguishing *Crtc1^-/-^* from wild-type mice. When considered separately, AUC for tCho was 0.783 (95% CI 0.709-0.858) and volume was 0.750 (95% CI 0.672-0.827). Analysis included samples from longitudinal (3 time points) and treatment (3 time points) studies for *Crtc1^-/-^* (n=22) and wild-type (n=31) mice. **(G)** ROC curves for energy metabolites lactate (Lac; red) and phosphocreatine (PCr, orange) and the average of the z-scores of both measurements (Lac+PCr; green) in dorsal hippocampus (DH) to distinguish mice with high versus low level of depressive-like behavior. Using a combination of both PCr and Lac provided an AUC of 0.656 (95% CI 0.555-0.756), while Lac had an AUC of 0.601 (95% CI 0.497-0.706) and PCr an AUC of 0.619 (95% CI 0.516-0.722), when considered separately. Analysis included samples from longitudinal (3 time points) and treatment (2 time points) studies for *Crtc1^-/-^* (n=22) and wild-type (n=31) mice.

## Supplemental Figure 5

**Figure S5: Supplementary ^13^C-labeling curves and metabolic flux values from mathematical modeling**

**(A)** Isotopic ^13^C-enrichment curves of remaining hippocampal metabolites included in the modeling (mean±s.d.) during ^1^H-[^13^C]-MRS experiment. Fitting of the data with a pseudo 3-compartment model of brain glucose metabolism is shown with a straight line for wild-type (WT; in blue) and *Crtc1*^-/-^ (in red) mice. **(B-C)** Metabolic fluxes (mean±s.d.) determined using a pseudo 3-compartment model (b) or a 1- compartment model (c) of brain glucose metabolism. Estimated parameters from the 1-compartment model of brain energy metabolism: Blood lactate influx V_dil_^in^; TCA cycle V_TCA_; transmitochondrial flux V_x_; neurotransmission flux V_NT_ and glial dilution factor V_dil_^g^. Estimated parameters from the pseudo 3-compartment model of brain energy metabolism (fluxes are separated into glutamatergic (^e^), GABAergic (^i^) and glial (^g^) compartments): The pyruvate dehydrogenase activity (V_PDH_), glial tricarboxylic acid cycle (V_g_), a dilution flux from blood lactate (V_dil_^in^) and from blood acetate (V_dil_^g^), a transmitochondrial flux (V_x_), a neurotransmission flux (V_NT_), pyruvate carboxylase flux (V_PC_), a Gln efflux (V_eff_), glutamine synthetase activity (V_GS_), glutamate decarboxylase activity (V_GAD_), GABA TCA shunt (V_shunt_) and two exchange fluxes between two Gln or two GABA pools (V_ex_^g^ and V_ex_^i^). Parameters calculated from these metabolic fluxes: inhibitory TCA cycle V_TCA_^i^; glial TCA cycle rate V_TCA_^g^; glutamine synthetase activity V_GS_; GABA shunt rate V_shunt_^i^(=V_shunt_^g^); lactate blood efflux V_dil_^out^; total TCA cycle or the oxidative cerebral metabolic rate of glucose CMR_Glc_(ox). Cerebral metabolic rate of glucose (CMR_Glc_) was used from the ^18^FDG-PET experiment. All the *P* values are from unpaired Student t-test, **P*<0.05, ***P*<0.005, ****P*<0.0005, *****P*<0.0001. Flux estimates are reported with the standard deviation generated by the MC simulation. All fluxes are given in µmol/g/min. **(D)**The ATP production (in µmol/g/min) was calculated with known yields (from Hertz et al(17).) for each energy pathway and compared between excitatory and inhibitory contributions. Consumption of one molecule of glucose produces 2 ATP and 2 NADH. Mitochondrial function produces ~23 ATP from the action of pyruvate dehydrogenase (PDH) and tricarboxylic acid (TCA) per pyruvate molecule. The malate-aspartate shuttle (MAS) fuels mitochondrial electron transport system (ETS) with cytoplasmic reducing equivalents, yielding ~2.5 ATP per molecule of NADH.

## Supplemental Table1
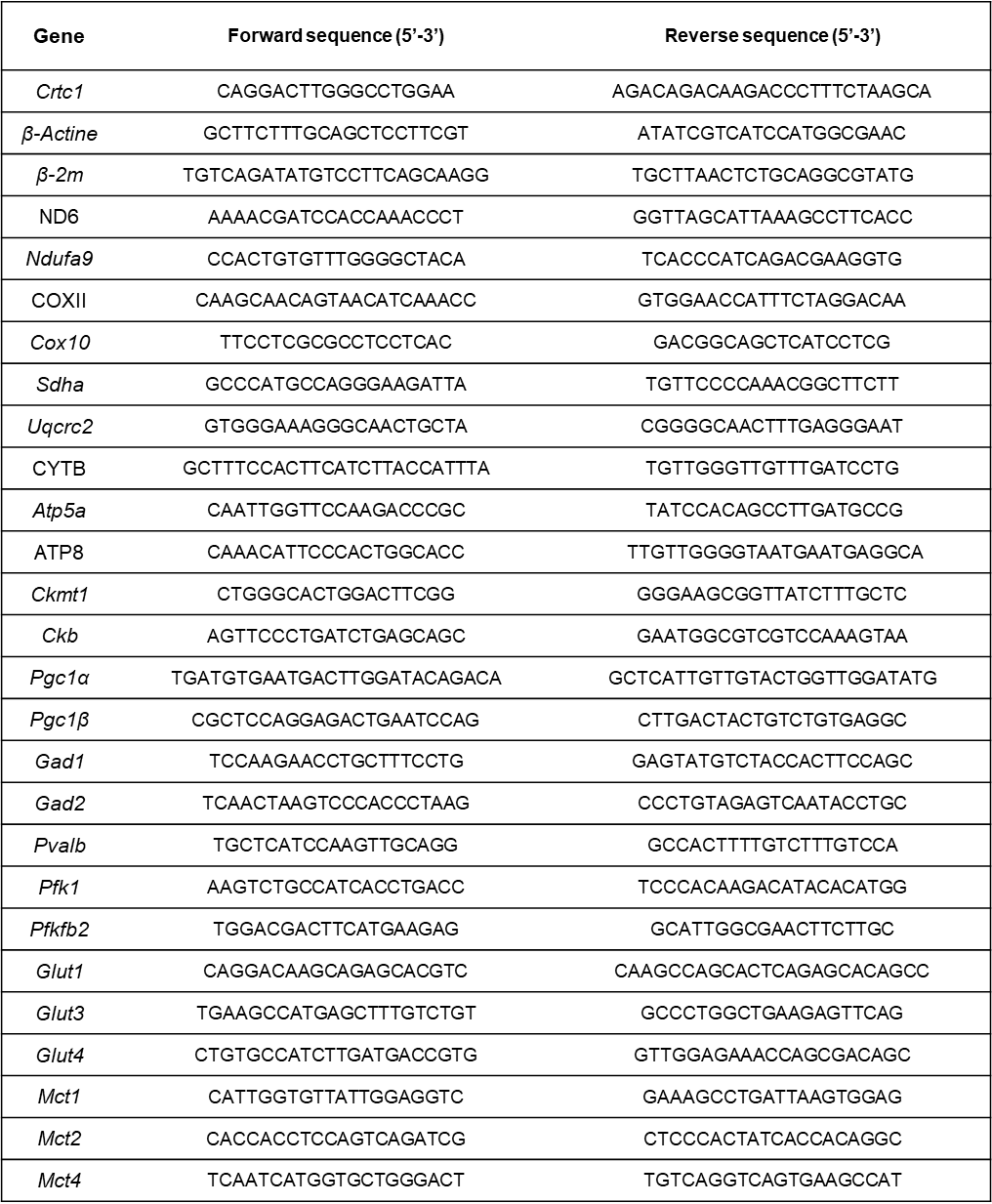


**Table S1: List of primers**

*Crtc1*: CREB Regulated Transcription Coactivator 1; β-2m: β_2_ microglobulin; ND6: Mitochondrially Encoded NADH:Ubiquinone Oxidoreductase Core Subunit 6; *Ndufa9:* NADH:ubiquinone oxidoreductase subunit A9; COXII: Cytochrome c oxidase subunit 2; *Cox10*: Cytochrome C Oxidase Assembly Factor Heme A:Farnesyltransferase COX10; *Sdha*: Succinate Dehydrogenase Complex Flavoprotein Subunit A; *Uqcrc2:* Ubiquinol-Cytochrome C Reductase Core Protein 2; CYTB: mitochondrially encoded cytochrome b; *Atp5a:* ATP synthase lipid-binding protein; ATP8: Mitochondrially Encoded ATP Synthase Membrane Subunit 8; *Ckmt1:* Creatine kinase U-type, mitochondrial; *Ckb:* Creatine kinase B-type; *Pgc1α:* Peroxisome Proliferator-Activated Receptor Gamma Coactivator 1-Alpha; *Pgc1* β*:* Peroxisome Proliferator-Activated Receptor Gamma Coactivator 1-Beta; *Gad1:* glutamate decarboxylase 1; *Gad2:* glutamate decarboxylase 2; *Pvalb:* Parvalbumin; *Pfk1:* ATP-dependent 6-phosphofructokinase subunit alpha; *Pfkfb2:* 6-phosphofructo-2-kinase/fructose-2,6-bisphosphatase 2; *Glut1-4:* facilitated glucose transporter, member 1-4; *Mct1-4:* monocarboxylic acid transporters, member 1-4.
